# Impact of Fixed Partial Dentures on Oral Microbial Flora and Gingival Health: An In Vitro Assessment of Salivary Microbial Profiles

**DOI:** 10.7759/cureus.65220

**Published:** 2024-07-23

**Authors:** Maaz Vohra, Vaishnavi Rajaraman, Amrutha Shenoy, Rithanya M

**Affiliations:** 1 Department of Prosthodontics and Implantology, Saveetha Dental College, Saveetha Institute of Medical and Technical Sciences, Saveetha University, Chennai, IND

**Keywords:** microscopic study, oral hygiene practice, anaerob microorganism, microbial colonization, fixed dental prosthesis

## Abstract

Introduction

The oral cavity hosts diverse microorganisms affected by factors like pH, temperature, and oxygen levels, influencing disease potential. Dentists manage oral diseases and improve aesthetics using durable restorations. Understanding periodontal response to crowns and fixed partial dentures (FPDs) is essential for effective treatment. This study aims to assess the impact of FPDs on periodontal health by comparing microbial colonies in individuals with and without FPDs. The hypothesis is that there will be no difference in microbial flora and gingival health between the two groups.

Materials and methods

This in vitro study utilized 40 salivary samples divided into two groups: 20 from patients with FPDs/crowns (Group 1) and 20 from patients without (Group 2). Unstimulated saliva was collected, diluted, and cultured on nutrient agar and Sabouraud Dextrose Agar to quantify anaerobic bacteria and *Candida *colonies. Colony counts were scored from 0 to IV based on colony-forming unit (CFU), and microscopic examination identified the types of microbes present. Data were analyzed using an unpaired t-test with IBM SPSS Statistics for Windows, Version 26 (Released 2019; IBM Corp., Armonk, New York, United States), with significance set at p < 0.05.

Results

The independent t-test analysis showed significantly higher mean CFUs of anaerobic microbes in Group 1 (experimental) compared to Group 2 (control) (p = 0.000). However, mean CFUs of *Candida* did not significantly differ between groups (p = 0.194). Microscopic examination identified *Enterococcus faecalis, Pseudomonas aeruginosa, Candida albicans, Staphylococcus aureus*,* *and* Streptococcus mutans* in the experimental group, whereas the control group contained only *Staphylococcus aureus *and* Streptococcus mutans.*

Conclusion

Individuals with FPDs exhibit higher concentrations of anaerobic microbes and specific bacteria, suggesting an increased risk of gingival inflammation and emphasizing the importance of maintaining good oral hygiene.

## Introduction

The oral cavity hosts a wide range of microorganisms due to its complex environment, which includes varying physical and chemical properties. Different areas within the mouth such as the tongue, teeth, gums, and mucous membranes harbor a variety of microbial populations. These populations are influenced by factors such as pH, temperature, redox potential, and oxygen levels, which affect microbial colonization and the potential for disease [1]. Over 250 microbial species, including key pathogens like *Actinobacillus actinomycetemcomitans, Tannerella forsythia, Porphyromonas gingivalis*,* *and *Streptococcus mutans* have been identified [2]. Dentists work to manage oral diseases while improving both the aesthetic and functional aspects of the mouth using durable and biocompatible restorations. It is important to comprehend the response of periodontal tissues to artificial crowns and fixed partial dentures (FPDs) in order to create effective treatment strategies. Evaluating each patient's needs is crucial for treatment planning, and a thorough analysis of the relevant evidence is vital for achieving reliable and predictable treatment outcomes [3].

It is well-established that the human oral cavity serves as a dynamic habitat for microorganisms. Evidence indicates that physiological changes in oral microbes occur throughout an individual's life. Fluctuations in microbial composition are significantly influenced by environmental factors. Additionally, the gradual decline in immune function with aging may impact the composition of oral microflora [4]. Clinical studies have shown that the oral microbiome evolves with age and have explored how indirect restorations impact periodontal tissues [5]. Research indicates that localized gingival inflammation can be caused by issues such as inadequate marginal adaptation, excessively deep margins, rough surfaces, and excessive contouring. This inflammation frequently arises because these restorations create a favorable environment for harmful microbes to thrive [6]. Multiple studies have shown that artificial crowns and FPDs can contribute to gingival inflammation [7]. Follow-up trials spanning 15 years found that teeth with crowns or FPDs had average probing depths that were 0.05 to 0.5 mm greater than those of control teeth [8]. Longitudinal studies conducted over 15 years have documented attachment loss after crown placement, with average losses ranging from 0.15 to 1.3 mm throughout the study periods [9]. Furthermore, several studies observed alveolar bone resorption in controlled periodontal patients, occurring at rates of 0.03 to 0.07 mm per year. Hence, it is crucial to use high-quality crowns and FPDs to reduce the risk of gingival inflammation [10].

Studies have investigated the effects of indirect restorations on periodontal health by measuring gingival index scores, radiographic bone levels, and probing depths [11]. Many of the studies employ similar methods to evaluate the effect of artificial crowns or FPDs on periodontal health [12]. This research offers a unique approach by examining microbial colonies in saliva, thus assessing the periodontal health. Previous research has explored microbial colonization on dentures using in vitro models like microtiter plates, acrylic strips, and discs [13]. Although most studies focus on complete or removable dentures, this in vitro research aims to assess gingival health by comparing and evaluating the types of microbial flora in the oral cavity of individuals with FPDs versus those without any crowns or FPDs. The null hypothesis for this study is that there will be no difference in the types of microbial flora between individuals with FPDs and those without, thus exhibiting no change in gingival health.

## Materials and methods

Study design

The in vitro study was conducted following approval from the Institutional Systematic Review Board (SRB/SDC/PROSTHO- 2105/24/110) and Institutional Human Ethical Committee (IHEC/SDC/PROSTHO-2105/24/110). This study utilized 40 salivary samples divided into two groups. Group 1 (the experimental group) consisted of samples from patients with FPDs or crowns that had been in the oral cavity for at least six months. Group 2 (the control group) included samples from patients without any FPDs or crowns. Each group contained 20 samples, making a total of 40 samples. A sample size calculation was performed using G*Power software (Heinrich-Heine-Universität Düsseldorf, Germany, Version 3.1). With a significance level (α) set at 0.05 and a power of 0.85, the confidence level was 0.95 (1 - α). An effect size of 0.8 was assumed based on previous studies with similar methodologies, leading to a required sample size of 20 samples per group, making a total of 40 samples [14].

Inclusion and exclusion criteria 

Participants were recruited from the outpatient department of our institution. Inclusion criteria were adults aged 20-60 years, with at least one FPD or crown in place for a minimum of six months for the experimental group and no FPDs or crowns for the control group. Exclusion criteria included current smokers, individuals with systemic conditions like diabetes and immunosuppressive diseases, those on antibiotics or antifungal treatment within the past three months, and individuals with poor oral hygiene practices. Age, sex, and oral hygiene habits were recorded, and only those with similar oral hygiene practices were included to control these confounding factors.

Sample preparation

**Figure 1:**
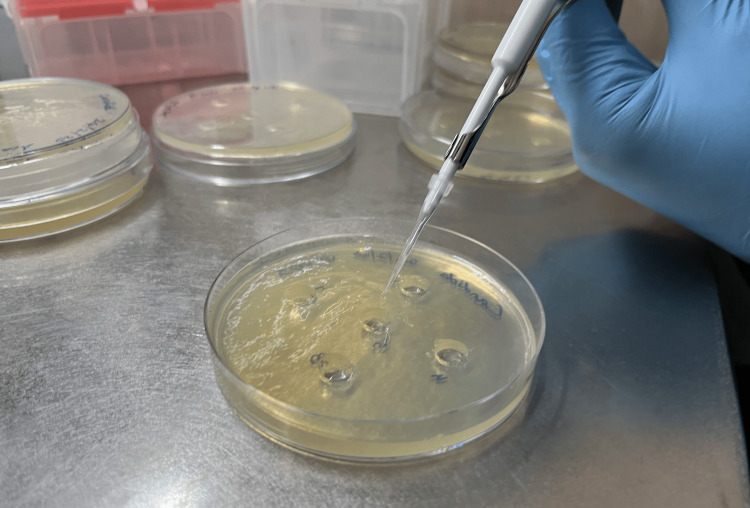
Inoculation of diluted sample

Participants were asked to avoid eating, drinking, or performing oral hygiene for at least two hours prior to saliva collection. Following this, unstimulated saliva was collected. The saliva was diluted with phosphate-buffered saline (PBS, pH 7.4) at a ratio of 1:10-8. Given that most salivary bacteria are anaerobes, the total number of anaerobic bacteria in the saliva was quantified. For this, 100 µL of each dilution was plated onto nutrient agar and incubated for one week at 37°C in an anaerobic environment with 80% N₂, 10% CO₂, and 10% H₂. To determine the number of *Candida* colonies, 100 µL of saliva was inoculated onto Sabouraud Dextrose Agar and incubated at 30°C for 48 hours in aerobic conditions (Figure 1). The total counts of anaerobic bacteria and *Candida *species were reported as colony-forming units (CFUs). The cultures were subsequently stained and examined under a microscope to identify the microbial species present.

Estimation of colonies

After 48 hours of incubation, the colonies were counted. The total number of colonies was calculated and multiplied by a factor based on the volume of the streaking loop. This procedure was standardized and applied to all culture plates. The following is the scoring system used: Score 0 suggested no growth. Score I was given for fewer than 1,000 CFUs. Score II corresponded to 1,000 to 10,000 CFU. Score III was assigned for 10,000 to 100,000 CFU. Finally, Score IV represented more than 100,000 CFU. 

Statistical analysis

Data collection and tabulation was done using Google Forms. The mean CFU values were analyzed by unpaired t-test using IBM SPSS Statistics for Windows, Version 26 (Released 2019; IBM Corp., Armonk, New York, United States), following an assessment of the dataset's normality. Statistical significance was set at 0.05 (p < 0.05).

## Results

Table 1 compares the key demographic and clinical characteristics between two groups: individuals with fixed partial dentures (FPD/crowns) and individuals without FPD/crowns.

**Table 1:** Characteristics of study populations M: Male; F: female; SD: standard deviation; FPD: fixed partial dentures; N/A: not applicable

Characteristic	Group 1 (FPD/crowns)	Group 2 (no FPD/crowns)	p-value
Sample size	20	20	-
Age (mean ± SD)	45.3 ± 10.2 years	42.8 ± 11.5 years	0.428
Gender (M/F)	12/8	10/10	0.527
Oral hygiene (good/fair/poor)	8/9/3	10/7/3	0.765
Duration of FPD use (mean ± SD)	8 ± 2 months	N/A	-

**Figure 2:**
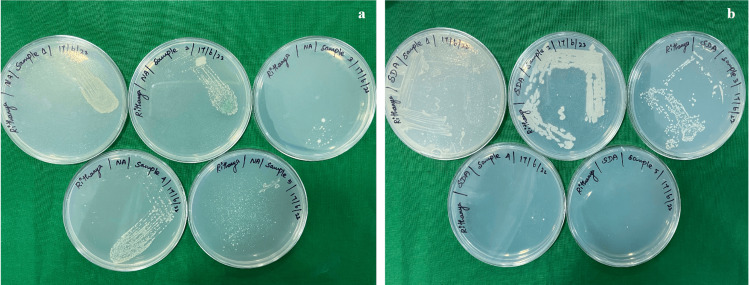
(a) Colonies of bacteria cultured on nutrient agar. (b) Colonies of fungi cultured on Sabouraud Dextrose Agar

**Figure 3:**
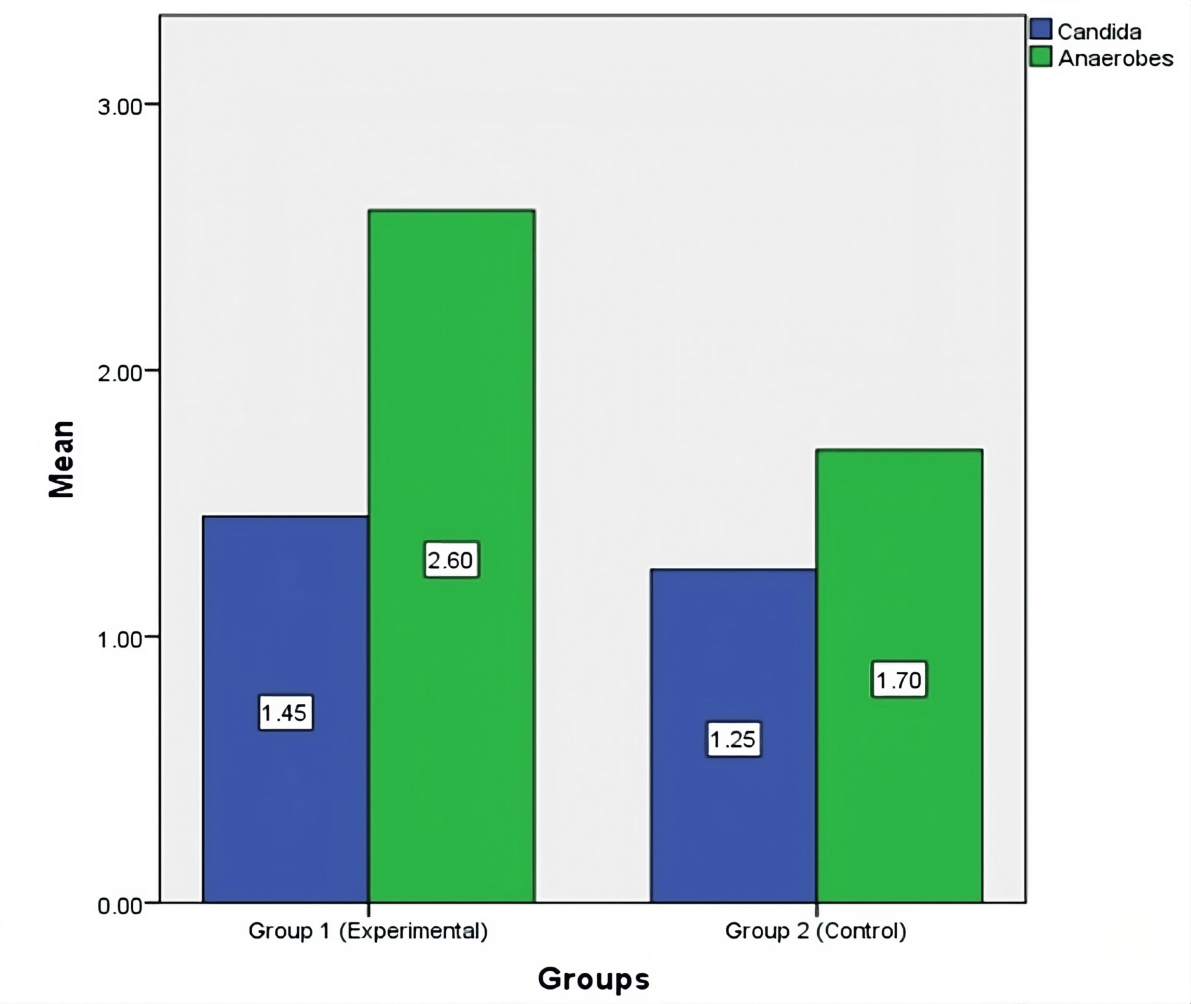
Graphical representation of mean colony-forming units in experimental and control groups

The mean CFUs of anaerobic microbes for two groups were analyzed using an independent t-test (Figure 2a, Table 2). Group 1 (experimental) and Group 2 (control) both have 20 samples. Group 1 has a mean CFU of 2.60 (SD = 0.680), and Group 2 has a mean CFU of 1.70 (SD = 0.656). The standard errors are 0.152 for Group 1 and 0.146 for Group 2. Both groups have a 95% confidence interval range from 0.471 to 1.328. The p-value is 0.000, indicating a statistically significant difference between the groups. This suggests that Group 1, the experimental group, has a higher concentration of anaerobic microbes compared to the control group (Figure 3).

**Table 2:** Mean colony-forming units (CFUs) of anaerobic microbes as observed on nutrient agar statistically analyzed used in independent t-test Group 1 is the experimental group, while Group 2 resembles the control group. *Suggests p-value < 0.05

Groups	N	Mean ± SD	Standard error	95% Confidence interval		p-value
				Lower	Upper	
Group 1	20	2.60 ± 0.680	0.152	0.471	1.328	0.000*
Group 2	20	1.70 ± 0.656	0.146	0.471	1.328	

The mean CFUs of *Candida *observed on Sabouraud Dextrose Agar for two groups, analyzed using an independent t-test (Figure 2b, Table 3). Group 1 (experimental) consists of 20 samples with a mean CFU of 1.450 (SD = 0.510) and a standard error of 0.114. Group 2 (control) also has 20 samples, with a mean CFU of 1.250 (SD = 0.444) and a standard error of 0.099. The 95% confidence interval for both groups ranges from -0.106 to 0.506. The p-value is 0.194, indicating no statistically significant difference between the two groups. This suggests that the concentration of *Candida *does not significantly differ between the experimental group and the control group (Figure 3).

**Table 3:** Mean colony-forming units (CFUs) of Candida as observed on Sabouraud Dextrose Agar statistically analyzed used in independent t-test Group 1 is the experimental group, while Group 2 resembles the control group

Groups	N	Mean ± SD	Standard error	95% Confidence interval		p-value
				Lower	Upper	
Group 1	20	1.450 ± 0.510	0.114	-0.106	0.506	0.194
Group 2	20	1.250 ± 0.444	0.099	-0.106		

**Figure 4:**
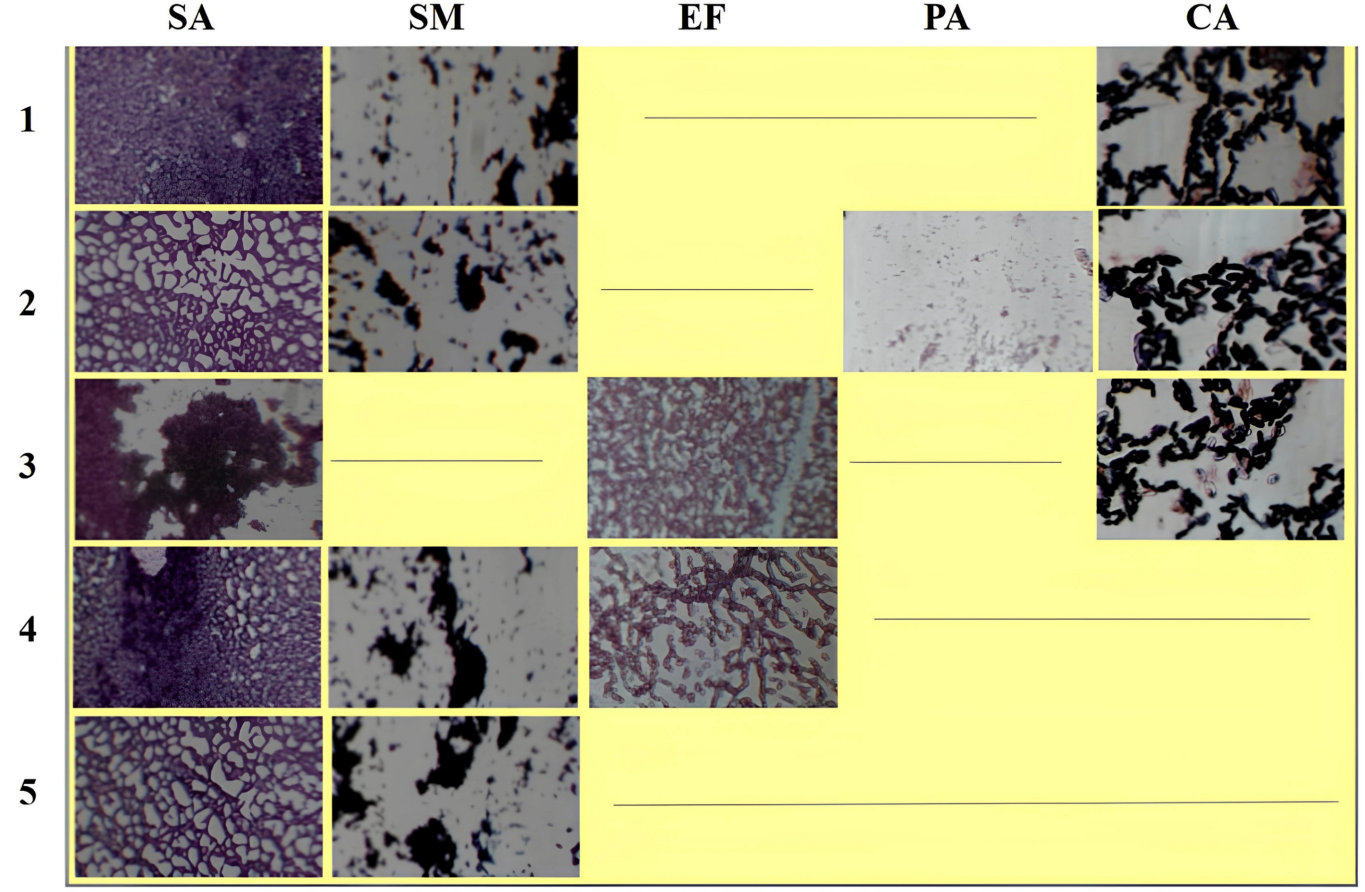
Microscopic examination of isolated microbes from samples 1-5. Sample 5 resembles the control group SA:* Staphylococcus aureus*;SM: Streptococcus mutans; EF: *Enterococcus faecalis*; PA: *Pseudomonas aeruginosa*; CA: *Candida albicans*

Microscopic examination revealed that the experimental group contained *Enterococcus faecalis, Pseudomonas aeruginosa, Candida albicans, Staphylococcus aureus*,* *and *Streptococcus mutans*. In comparison, sample 5, representing the control group, showed the presence of only *Staphylococcus aureus *and *Streptococcus mutans *(Figure 4).

## Discussion

The current experiment aimed to compare the microbial presence in individuals with FPDs or crowns (experimental group) to those without these restorations (control group). The findings revealed that the mean concentration of anaerobic microbes was significantly higher in the experimental group (mean CFU 2.60) compared to the control group (mean CFU 1.70) (p = 0.000). However, there was no significant difference in *Candida* CFUs between the two groups. Microscopic examination identified Staphylococcus aureus, Streptococcus mutans, Enterococcus faecalis, Pseudomonas aeruginosa, and* Candida albicans* in the experimental group, whereas only *Staphylococcus aureus *and *Streptococcus mutans* were found in the control group. Consequently, the null hypothesis was rejected, indicating that FPDs and crowns significantly influence the oral microbial profile, particularly anaerobic bacteria.

Research has consistently demonstrated that both removable and fixed prostheses significantly impact oral and gingival health. A notable 15-year clinical trial revealed that crowned teeth experienced higher levels of gingival inflammation, as evidenced by more frequent gingival index (GI) scores of 2 and 3 compared to noncrowned teeth [15]. However, a contrasting longitudinal study reported no significant difference in bone loss between crowned and control teeth, with both groups exhibiting similar horizontal bone loss rates of 0 to 1 mm or 1 to 2 mm [16]. Additionally, other studies have noted annual alveolar bone resorption rates of 0.03 to 0.07 mm in patients with controlled periodontal conditions, suggesting that crowns do not significantly affect bone loss [17]. The role of microbial species such as *Streptococcus *and* Staphylococcus* is crucial in understanding gingival health.* Streptococcus* species, particularly *Streptococcus mutans*, are key contributors to dental plaque and biofilm formation on teeth and gums, which can lead to gingival inflammation. Conversely, *Staphylococcus aureus*, although less commonly found in dental biofilms, can cause localized infections that exacerbate gingival inflammation. The presence and activity of these microbes can disrupt the balance of the oral microbiome, leading to increased inflammation and potentially worsening periodontal health. A study comparing patients with removable or fixed prostheses to those with gingivitis, but no prostheses identified a total of 82 bacterial species across both groups. Of these, 27 species were common to both groups, 29 species were found exclusively in patients with prostheses, and 26 species were present only in individuals with gingivitis who did not use prostheses [18]. *Candida* species were found in both groups, suggesting that prosthetic use doesn’t significantly alter the *Candida *profile compared to individuals with gingivitis. This indicates a similar microbial profile between patients with FPDs and those with gingivitis, illustrating the complex relationship between prosthetic use and oral microbial communities. The lack of significant difference in *Candida* CFUs may be due to several reasons. *Candida*, being an opportunistic pathogen, can thrive under various conditions, but its growth might not be as affected by FPDs as anaerobic bacteria are. This highlights the need to understand how microbial dynamics impact gingival inflammation and periodontal disease. Regular oral hygiene and dental checkups are crucial to minimize the negative effects of prosthetics on oral health ​​​​[19,20].

This study, like any scientific research, has its limitations. To address the heterogeneity within groups, future studies should incorporate additional statistical analyses to control for confounding factors such as systemic health and smoking habits. Variability in these factors could significantly impact gingival health and microbial profiles. The current study did not account for these variables, which could have influenced the results. For instance, systemic conditions like diabetes or cardiovascular disease and varying oral hygiene practices may alter microbial colonization and periodontal health independently of the presence of FPDs. Future research should focus on examining bacterial complexes and their interactions in individuals with FPDs, considering factors like prosthesis type, bonding materials, and oral hygiene practices. This approach will provide a more comprehensive understanding of how these variables influence gingival health and microbial dynamics. Emphasizing rigorous oral hygiene and regular dental checkups remains crucial for mitigating the adverse effects of prosthetic use on oral health. Addressing these factors will enhance the robustness of future studies and contribute to a deeper understanding of the complex relationship between dental prostheses and oral health.

## Conclusions

This study revealed that individuals with FPDs have a significantly higher concentration of anaerobic microbes compared to those without FPDs. Although *Candida* levels were similar between the two groups, *Staphylococcus aureus* and *Streptococcus mutans* were more prevalent among those with FPDs. These findings suggest an increased likelihood of gingival inflammation in individuals with FPDs. The results highlight how FPDs affect oral microbial communities and assert the importance of maintaining good oral hygiene. 
